# ctDNA: An emerging neoadjuvant biomarker in resectable solid tumors

**DOI:** 10.1371/journal.pmed.1003771

**Published:** 2021-10-12

**Authors:** Christopher Abbosh, Charles Swanton

**Affiliations:** 1 Cancer Research UK Lung Cancer Centre of Excellence, University College London Cancer Institute, London, United Kingdom; 2 Cancer Evolution and Genome Instability Laboratory, The Francis Crick Institute, London, United Kingdom

## Abstract

Christopher Abbosh and Charles Swanton discuss circulating tumor DNA as a potential biomarker for neoadjuvant treatment response in solid tumors.

## Introduction

Three studies presented within this special issue of PLOS Medicine focus on evaluation of circulating tumor DNA (ctDNA) as a response biomarker in early-stage solid tumours. Both Yaqi Wang and Pradeep Chauhan and their respective colleagues evaluate ctDNA as a tool capable of predicting complete pathological response (pCR) in locally advanced rectal cancer (LARC) and muscle invasive bladder cancer (MIBC), respectively [1,2]. Jeanne Tie and colleagues focus on ctDNA evaluation in high-risk metastatic colorectal cancer with liver metastases (CRLM), both during neoadjuvant therapy and following surgery and adjuvant therapy [3]. In this brief perspective we evaluate these advances within the wider context of recently published work.

## ctDNA as a neoadjuvant response biomarker

Quantitation of ctDNA kinetics over time can act as a dynamic biomarker of tumour response to targeted therapies, immunotherapy and radiation therapy [4–6]. In a neoadjuvant setting ctDNA kinetics could guide escalation of neoadjuvant therapy in non-responders, or be used as a de-escalation tool to curtail the number of neoadjuvant cycles being administered or reduce the need for further therapy (including surgery).

In relation to the latter point, Wang and colleagues draw attention to the potential for deferral of surgery in patients exhibiting complete clinical response (cCR; i.e., no clinical, endoscopic, or radiographic evidence of disease) following neoadjuvant chemoradiation treatment for LARC [2]. This is termed a “watch and wait” strategy. In patients exhibiting cCR following neoadjuvant treatment, probability of LARC recurrence without surgical intervention is low [7]. However, cCR is an imperfect surrogate for pCR therefore the authors sought to determine whether ctDNA evaluation during neoadjuvant therapy could improve the accuracy of delineating neoadjuvant treatment response. Through evaluation of a metric termed T234_clearance (describing absence of the highest mutant allele frequency baseline mutation at all 3 pre-operative timepoints, i.e., persistent ctDNA clearance during neoadjuvant treatment) the team identified 20 of 89 patients who lacked evidence of T234_clearance following neoadjuvant treatment. Four of these patients exhibited cCR based on Magnetic Resonance Imaging (MRI) evaluation [2]. This observation highlighted some discordance between cCR and ctDNA clearance kinetics. Within the T234_clearance negative, cCR positive population, one of four patients suffered disease recurrence. This suggests that ctDNA clearance could refine cCR evaluation in LARC [2]. An exploratory analysis demonstrated that combining multiple ctDNA features alongside MRI response information in a prediction model improved discrimination of pCR from non-pCR, compared to ctDNA features or MRI response parameters alone [2].

The study from Wang and colleagues supports prior findings from Murahashi and colleagues who also evaluated post-treatment ctDNA kinetics in patients undergoing neoadjuvant treatment for LARC [8]. In Murahashi and colleagues’ analysis, post-treatment ctDNA levels decreased in 11 of 12 patients who experienced response to neoadjuvant treatment (either pCR or 12 months relapse free if watch and wait strategy adopted); in contrast, 7 of 39 patients who did not experience clinical response displayed increase in ctDNA levels [8]. Combining decrease in ctDNA during treatment with endoscopic complete response evaluation improved neoadjuvant therapy response stratification in this study [8]. Overall, these data suggest that monitoring ctDNA following neoadjuvant treatment for LARC has potential as a complementary tool to improve accuracy of current cCR measures.

Like the application of ctDNA in LARC, Chauhan and colleagues asked whether evaluation of urinary ctDNA (utDNA) could differentiate pCR from non-pCR in patients being treated with neoadjuvant therapy for MIBC [1]. Development of an alternative to pCR in this setting could avoid surgical cystectomy and urinary diversion in excellent prognosis patients. The team identified that quantifying utDNA on the basis of non-silent mutations (mutations that result in a change to an encoded amino acid sequence), but not silent mutations (mutations that do not change the amino acid sequence), accurately classified pCR versus non-pCR and suggested that field-effect within bladder urothelium could underlie this difference (a field-effect or field cancerisation describes tissue that has been pre-conditioned by carcinogen exposure, facilitating the process toward cancer formation [9]). Excluding silent mutations, a utDNA minimal residual disease threshold was optimised based on analyses of healthy participants and non-pCR patients. Application of this threshold to the cohort revealed a sensitivity of 81% and a specificity of 81% for non-pCR prediction. Based on these findings Chauhan and colleagues suggest utDNA could be used to complement emerging clinical predictors of cPR, such as MRI-based response criteria, and draw attention to an Alliance for Clinical Trials in Oncology Study on non-metastatic MIBC (clingov: NCT03609216) which will provide a framework for validation of the team’s observations. This work highlights the potential for utDNA to be utilised as a biomarker in bladder cancer, building upon previous work using the same ctDNA platform (uCAPP-seq) by Dudley and colleagues that demonstrated utDNA as capable of identifying localised early-stage bladder cancer and tracking recurrent disease following local bladder cancer treatment [10].

Tie and colleagues explored ctDNA as a curative-therapy response biomarker in CRLM. Within a cohort of patients who underwent neoadjuvant chemotherapy, they noted a median 40.93-fold decrease in ctDNA levels during treatment with 13 of 18 evaluable patients exhibiting absence of ctDNA detection pre-cycle 4 of treatment: all 4 patients with pCR in the resection specimen experienced ctDNA clearance prior to cycle 3 or 4 of neoadjuvant treatment. However, ctDNA clearance during neoadjuvant chemotherapy had no impact on 5-year relapse free survival (RFS) when compared to lack of ctDNA clearance. In contrast, the team identified that ctDNA detection after surgery was a strong predictor of reduced RFS, with patients who were ctDNA positive following curative therapy (surgery +/- adjuvant therapy) exhibiting a 5-year RFS rate of 0% versus 75.6% in ctDNA negative patients. These data highlight the importance of associating ctDNA clearance dynamics during neoadjuvant treatment with post-operative survival endpoints, since in this study ctDNA clearance with neoadjuvant chemotherapy did not translate into reduced risk of disease recurrence following surgery.

Supporting ctDNA as a neoadjuvant response biomarker in other tumor types, data in non-small-cell lung cancer (NSCLC) from the neoadjuvant CheckMate-816 study, a randomized, phase III study comparing neoadjuvant platinum chemotherapy with or without nivolumab in stage IB-IIIA NSCLC, highlighted that ctDNA clearance at day 1 cycle 3 post-combination chemotherapy and immune checkpoint inhibitor treatment associates with pCR [11]. Stage II-III early-stage breast cancer patients treated with either standard neoadjuvant chemotherapy or neoadjuvant chemotherapy plus MK-2206 (an AKT inhibitor) underwent longitudinal ctDNA-analyses using a tumour-informed assay in the I-SPY2 platform trial [12]. Absence of ctDNA clearance following cycle 1 of therapy associated with an increased likelihood of non-pCR (24 of 29 [83%] patients with non-pCR had residual ctDNA detected post cycle 1 of therapy versus 14 of 27 [52%] who cleared ctDNA post cycle 1 of therapy [12]). In this study the authors categorised patients by pCR status and ctDNA status after neoadjuvant therapy and identified that patients who were ctDNA negative but did not achieve pCR had a similar risk of metastatic recurrence compared with patients who did achieve pCR, suggesting that ctDNA clearance could divide non-pCR patients into high- and low-risk categories [12].

In conclusion, the findings presented in this special issue add to an emerging literature highlighting a need to explore the translational potential for ctDNA assessment as a response biomarker in the neoadjuvant setting. These data are particularly relevant in LARC and MIBC where treatment response biomarkers that are not reliant on pathological examination of resection specimens are required to guide non-operative management decisions. The data from Tie and colleagues suggest that the capability of neoadjuvant chemotherapy-induced ctDNA clearance to act as a surrogate of long-term survival benefit from curative intent therapy could be absent in CLRM, however the sample size in this study was modest which may have limited ability to detect an association. It is conceivable that the utility of ctDNA as a neoadjuvant response biomarker may vary by therapeutic class and solid tumour type. To address this issue, it will be important for prospective interventional trials to incorporate ctDNA clearance kinetics as an endpoint to determine surrogacy of these measures for survival across solid-tumour types. Finally, to gain understanding of the relative merits and disadvantages of ctDNA-based response metrics versus conventional clinical measures of response (such as endoscopic and imaging-based evaluations), direct comparison of ctDNA clearance with these approaches is warranted.
